# NEK1 Phosphorylation of YAP Promotes Its Stabilization and Transcriptional Output

**DOI:** 10.3390/cancers12123666

**Published:** 2020-12-07

**Authors:** Md Imtiaz Khalil, Ishita Ghosh, Vibha Singh, Jing Chen, Haining Zhu, Arrigo De Benedetti

**Affiliations:** 1 Department of Biochemistry and Molecular Biology, LSU Health Sciences Center, Shreveport, LA 71130, USA; mkhal2@lsuhsc.edu (M.I.K.); ighosh@lsuhsc.edu (I.G.); Vibha.Singh@utdallas.edu (V.S.); 2 Department of Molecular and Cellular Biochemistry and Proteomics Core, Center for Structural Biology, University of Kentucky, Lexington, KY 40506, USA; jchen4@email.uky.edu (J.C.); haining@uky.edu (H.Z.)

**Keywords:** tousled-like kinase (TLK), NIMA-related kinase 1 (NEK1), yes-associated protein 1 (YAP1), thioridazine (THD), MS-determined phosphopeptides

## Abstract

**Simple Summary:**

We earlier described the involvement of the TLK1>NEK1>ATR>Chk1 axis as a key determinant of cell cycle arrest in androgen-dependent prostate cancer (PCa) cells after androgen deprivation. We now report that the TLK1>NEK1 axis is also involved in stabilization of yes-associated protein 1 (YAP1), the transcriptional co-activator in the Hippo pathway, presumably facilitating reprogramming of the cells toward castration-resistant PCa (CRPC). NEK1 interacts with YAP1 physically resulting in its phosphorylation of 6 residues, which enhance its stability and activity. Analyses of cancer Protein Atlas and TCGA expression panels revealed a link between activated NEK1 and YAP1 expression and several YAP transcription targets.

**Abstract:**

Most prostate cancer (PCa) deaths result from progressive failure in standard androgen deprivation therapy (ADT), leading to metastatic castration-resistant PCa (mCRPC); however, the mechanism and key players leading to this are not fully understood. While studying the role of tousled-like kinase 1 (TLK1) and never in mitosis gene A (NIMA)-related kinase 1 (NEK1) in a DNA damage response (DDR)-mediated cell cycle arrest in LNCaP cells treated with bicalutamide, we uncovered that overexpression of wt-NEK1 resulted in a rapid conversion to androgen-independent (AI) growth, analogous to what has been observed when YAP1 is overexpressed. We now report that overexpression of wt-NEK1 results in accumulation of YAP1, suggesting the existence of a TLK1>NEK1>YAP1 axis that leads to adaptation to AI growth. Further, YAP1 is co-immunoprecipitated with NEK1. Importantly, NEK1 was able to phosphorylate YAP1 on six residues in vitro, which we believe are important for stabilization of the protein, possibly by increasing its interaction with transcriptional partners. In fact, knockout (KO) of NEK1 in NT1 PCa cells resulted in a parallel decrease of YAP1 level and reduced expression of typical YAP-regulated target genes. In terms of cancer potential implications, the expression of NEK1 and YAP1 proteins was found to be increased and correlated in several cancers. These include PCa stages according to Gleason score, head and neck squamous cell carcinoma, and glioblastoma, suggesting that this co-regulation is imparted by increased YAP1 stability when NEK1 is overexpressed or activated by TLK1, and not through transcriptional co-expression. We propose that the TLK1>NEK1>YAP1 axis is a key determinant for cancer progression, particularly during the process of androgen-sensitive to -independent conversion during progression to mCRPC.

## 1. Introduction

The founding member of the NIMA (never in mitosis gene A) family of protein kinases was originally identified in *Aspergillus nidulans* as a protein kinase essential for mitosis [1], and expression of a dominant-negative mutant of NIMA results in G2 arrest in vertebrate cells [2]. NIMA-related kinases (NEKs) have adapted to a variety of cellular functions in addition to mitosis [3]. In human cells, 11 NEKs were identified that are involved in several functions. For example, NEK2 is critical for centrosome duplication [3], whereas NEK6, 7, and 9 are regulators of the mitotic spindle and cytokinesis [4]. NEK1, NEK4, NEK8, NEK10, and NEK11 have been linked to the DNA damage response (DDR) and DNA repair pathways as well as ciliogenesis [3]. NEK1 mediates Chk1 activation likely by modulating the ATRIP/ATR interaction and activity [5], although this may be controversial [6]. NEK1 activity and relocalization to nuclei were reported to increase upon a variety of genotoxic stresses [5,7]. A defect in DNA repair in NEK1-deficient cells is suggested by the persistence of Double Strand Breaks (DSBs) after low-dose ionizing radiation (IR). NEK1-deficient cells fail to activate the checkpoint kinases Chk1 and Chk2, and fail to arrest properly at G1/S- or G2/M-phase checkpoints after DNA damage [8]. NEK1-deficient cells suffer major errors in mitotic chromosome segregation and cytokinesis, and become aneuploid [9]. Genomic instability is also manifested in NEK1^+/−^ mice, which later in life develop lymphomas with a higher incidence than wild type littermates [9]. NEK1 is also known to negatively regulate apoptosis by phosphorylating VDAC1, regulating the closure of the anion channel of the mitochondrial membrane, which promotes survival of renal cell carcinoma [10,11,12]. Loss of function mutation of NEK1 leads to DNA damage accumulation in the motorneurons that may lead to several neurodegenerative diseases such as amyotrophic lateral sclerosis (ALS) [13,14]. NEK1 is associated with primary cilia and centrosomes [15,16], which was reported to be implicated in the development of polycystic kidney disease (PKD) when there is a NEK1 deficiency [17]. However, the precise mechanism leading to PKD due to NEK1 insufficiency is not clear, but a clue came from the discovery that NEK1 interacts with and phosphorylates TAZ, involved in the E3 ligase complex, which regulates the stability of polycystin 2 [18]. TAZ is also a paralog of yes-associated protein (YAP), a transcriptional coactivator that mediates many functions in normal development and in disease pathology, such as cancer progression, including prostate cancer [19,20,21,22].

We recently uncovered a new DDR axis involving the protein kinase tousled-like kinase (TLK)1 as an early mediator of the DDR. TLK1 serves as an upstream activator of NEK1>ATR>Chk1 [6,23], which has important implications during the early stages of prostate cancer (PCa) progression to androgen independence (AI) [24,25]. We found that overexpression of wt-NEK1 (but not the T141A kinase-hypoactive mutant that cannot be phosphorylated by TLK1) hastens the progression of LNCaP cells to androgen-independent growth [24]. The protective cell cycle arrest mediated by the TLK1>NEK1 DDR pathway seems insufficient to explain the rapid growth recovery observed in bicalutamide-treated cells when NEK1 is overexpressed, and suggests that NEK1 may have additional functions. We suspected that it may regulate the Hippo pathway, as it was reported that ectopic expression of YAP is sufficient to convert LNCaP cells from androgen-sensitive (AS) to AI in vitro [19]. NEK1 was also found to phosphorylate TAZ specifically at S309 [18], and this was related to increased CTGF expression (one of TAZ/YAP transcriptional targets). TLKs may regulate the Hippo pathway through their activity on NEK1 upstream of YAP/TAZ. YAP/TAZ (60% identical) are the main effectors of the Hippo signaling pathway. This pathway is involved in regulating organ size through controlling multiple cellular functions including cell proliferation and apoptosis [26]. The Hippo pathway responds to a variety of signals, including cell–cell contact, mechano-transduction [21], and apico–basal polarity [20,26]. When the Hippo pathway is activated, kinases MST1/2 and LATS1/2 phosphorylate and inactivate YAP and TAZ. YAP and TAZ are transcriptional co-activators but lack DNA binding activity. Upon phosphorylation by MST and LATS kinases, they are sequestered in the cytoplasm, ubiquitylated by the β-TrCP ubiquitin ligase, and marked for proteasomal degradation (reviewed in [20]). YAP/TAZ are usually inhibited by cell–cell contact in normal tissues [26], while over-activation of YAP/TAZ through aberrant regulation of the Hippo pathway has been noted in many types of tumors. This is associated with the acquisition of malignant traits, including resistance to anticancer therapies; maintenance of cancer stem cells; distant metastasis [26]; and, in prostate, adenocarcinoma progression [27,28]. When the Hippo core kinases are “off”, YAP/TAZ translocate into the nucleus, binds to TEAD1–4, and activates the transcription of TEAD downstream target genes, leading to multiple oncogenic activities, including loss of contact inhibition, cell proliferation, epithelial–mesenchymal transition, and resistance to apoptosis. In PCa, YAP has been identified as an Androgen Receptor-binding partner that colocalizes with AR in both androgen-dependent and androgen-independent manners in castration-resistant PCa (CRPC) patients [27]. YAP is also found to be upregulated in AI-LNCaP-C4-2 cells and, when expressed ectopically in LNCaP cells, it activates AR signaling and confers castration resistance. Knockdown of YAP greatly reduces the rates of migration and invasion of LNCaP, and YAP-activated androgen receptor signaling is sufficient to promote LNCaP cells from an AS to an AI state in vitro, while YAP conferred castration resistance in vivo [19]. It was also recently determined that ERG (and the common *TMPRSS2*–*ERG* fusion) activates the transcriptional program regulated by YAP1, and that prostate-specific activation of either ERG or YAP1 in mice induces similar transcriptional changes and results in age-related prostate tumors [29]. However, it has remained unclear as to what the upstream activators of the Hippo pathway are in PCa, and we show in this report that TLKs have a role in this process via activation and induced stabilization of YAP from elevated phosphorylation by NEK1.

## 2. Materials and Methods

### 2.1. Plasmids and Antibodies

Wild type human full length NEK1 mammalian expression plasmid was purchased from Origene (MR216282). NEK1 T141A variant was generated by site-directed mutagenesis, as previously described [23]. Generation of His-tagged N-terminal NEK1 (aa 1–480) bacterial expression plasmid was conducted as previously described. Human full length MK5 bacterial expression plasmid was purchased from Vector Builder. The following antibodies were used in this study: mouse anti-YAP (Santa Cruz Biotechnology, SCBT, Dallas, TX, USA, cat# sc101199), rabbit anti-phospho-YAP (Cell Signaling Technology, CST, Dallas, TX, USA, cat# 13008), mouse anti-NEK1 (SCBT, cat# sc 398813, Dallas, TX, USA), rabbit anti-phospho-NEK1 pT141 (lab-generated), rabbit anti-phospho-tyrosine (CST, cat# 8954S, Dallas, TX, USA), HRP-conjugated anti-β-tubulin (SCBT, Dallas, TX, USA, cat# sc-23949), mouse IgG (SCBT, Dallas, TX, USA, cat# sc-2025), and rabbit anti-actin (Abcam, Cambridge, MA, USA, cat# ab1801).

### 2.2. Cell Culture

Human embryonic kidney HEK293 and HeLa cells were cultured in Dulbecco Modified Eagle Medium (DMEM) supplemented with 10% Fetal Bovine Serum (FBS) and 1% penicillin/streptomycin. HEK293T cells were cultured in D10 medium containing 10% FBS, 0.25% penicillin/streptomycin, and 1% glutamine in DMEM media. LNCaP cells were cultured in Roswell Park Memorial Institute (RPMI) 1640 supplemented with 10% FBS and 1% penicillin/streptomycin. NT1 cells were a kind gift from Dr. Xiuping Yu (Department of Biochemistry, Louisiana State University Health Sciences Center Shreveport) and cultured according to the published literature [30]. All other cells were purchased from American Type Culture Collection (ATCC). All the cells were maintained in a humidified incubator at 37 °C with 5% CO_2_.

### 2.3. Cell Treatment

LNCaP or HeLa or NT1 cells were plated as 5 × 10^5^ cells per well in a 6-well plate and grown until 70–80% confluency. Cells were treated with either 10 µM of either bicalutamide (Selleckchem, Houston, TX, USA, cat# S1190), thioridazine (THD; Sigma Aldrich, St. Louis, MO, USA, cat# T9025 or J54 [31], or in combination with both bicalutamide and THD for 24 h. After the treatment, cells were harvested for Western blotting (WB) analysis or qPCR analysis.

### 2.4. Cell Transfection

LNCaP cells were transfected with either wild type mouse full-length NEK1 or NEK1 T141A variant, as previously described [23]. TLK1 shRNA (ATTACTTCATCTGCTTGGTAGAGGTGGCT) was obtained from origene (Rockville, MD, USA, cat# TR320623). HeLa cells were plated as 10^5^ cells per well in a 6-well plate 24 h before shRNA transfection. Transfection was conducted using 140 nM and 280 nM of TLK1 shRNA by lipofectamine 3000 (Thermo Scientific, Waltham, MA, USA, cat# L3000-015) reagent for 24 h, following the manufacturer’s protocol, and subsequently selected the cells with 1 µg/mL of puromycin for 7 days. Puromycin-selected cells were harvested and knockdown efficiency was determined by WB.

### 2.5. Co-immunoprecipitation (co-IP)

Cells were lysed by sonication in 1X RIPA lysis buffer (SCBT, Dallas, TX, USA, cat# 24948). A total of 50 µL of equilibrated protein A/G agarose (SCBT, Dallas, TX, USA, cat# sc-2003) was incubated with either mouse anti-NEK1 antibody or mouse IgG antibody at 4 °C for 4 h with rotation. A total of 500 µg of protein lysate was added to the reaction and incubated overnight at 4 °C. Beads were washed thrice and eluted with 25 µL of 2X SDS-Laemmli buffer, and the entire volume was loaded into SDS-PAGE gel for WB analysis.

### 2.6. Generation of NT1 NEK1 Knockout (KO) Cells Lines

NT1 NEK1 KO clones were generated by lentiviral infection using NEK1 CRISPR gRNA (AAGGAGAGAAGTTGCTGTAT) cloned into pLentiCRISPR V2 vector backbone from Genscript (Piscataway, NJ, USA). Lentivirus containing NEK1 CRISPR gRNA was packaged using HEK293T cells. NT1 cells were infected with lentivirus using polybrene transfection reagent following standard protocol. After 72 h of infection, cells were supplemented with fresh media and selected with 1–2 µg/mL of puromycin for 10 days. To generate a single clonal population of NEK1 KO cells, we seeded 1–2 cells per well in a 96-well plate and grew them until confluency, and then transferred them to a bigger dish for expansion. KO efficiency was measured by Western blotting (WB) using anti-NEK1 mouse antibody from Santa Cruz Biotechnology (Dallas, TX, USA, cat# sc-398813).

### 2.7. Protein Purification

Recombinant His-tagged full-length MK5 and His-tagged NEK1 N-terminal-truncated proteins (NEK1ΔCT) were purified by affinity chromatography. Both MK5 and NEK1∆CT were transformed into Rosetta2 DE3 strain [23]. Expression of His-MK5 was induced with 1mM Isopropyl β- d-1-thiogalactopyranoside (IPTG) at 37 °C for 3–4 h, and His-NEK1ΔCT expression was induced with 0.5 mM IPTG overnight at 25 °C. Bacteria were pelleted down; dissolved in buffer containing 50 mM sodium phosphate (Na_2_HPO_4_ + NaH_2_PO_4_) of pH 8.0, 300 mM NaCl, 20 mM imidazole, and 1mM phenylmethylsulfonyl fluoride (PMSF); and lysed by sonication. Supernatants were incubated with Ni-NTA agarose (Qiagen, cat# 30210), and protein was eluted in buffer containing 50 mM sodium phosphate (Na_2_HPO_4_ + NaH_2_PO_4_) of pH 8.0, 300 mM NaCl, and 250 mM imidazole. Eluted proteins were dialyzed overnight at 4 °C using dialysis buffer containing 20 mM sodium phosphate (Na_2_HPO_4_ + NaH_2_PO_4_) of pH 7.7, 1 M NaCl, 10 mM β-mercaptoethanol, 0.5 mM ethylenediaminetetraacetic acid (EDTA) of pH 8.0, and 5% glycerol. After the dialysis, protein samples were run in SDS-PAGE gel to check their purity and correct molecular weight.

### 2.8. ADP Hunter Assay

ADP hunter assays were conducted to determine the catalytic activity of the purified kinases by the fluorescence detection of ATP to ADP conversion using an ADP Hunter Plus Assay kit (Eurofins, DeSoto, TX, USA, cat# 90-0083). Increasing amount of purified recombinant NEK1 or MK5 were incubated with either dephosphorylated α-casein (substrate for NEK1, source: Sigma-Aldrich, St. Louis, Missouri, USA, cat# C8032) or purified recombinant HSP27 (substrate for MK5, source: Abcam, Cambridge, MA, USA, cat# ab48740). The manufacturer provided kinase buffer, and 50 μM of ATP was added to the reaction, incubating the reaction at 30 °C for 30 min. Afterwards, reagent A and B were added sequentially, incubating the reaction at room temperature for 30 min. Stop solution was added and fluorescence intensity signal was measured at 530/590 nm excitation/emission wavelength. ADP concentration was determined by the standard curve through the serial dilutions of the ADP standards provided with the kit.

### 2.9. In Vitro Kinase Assay

In vitro kinase (IVK) assays were performed using purified recombinant proteins, kinase buffer, ATP, and/or [γ-^32^P] ATP. Purified recombinant GST-tagged YAP1 (Novus Biologicals, cat# Centennial, CO, USA, H00010413-P01) was incubated with either purified recombinant His-NEK1ΔCT or purified recombinant His-tagged MK5. Kinase buffer (10X) contains 10 mM Tris-Cl of pH 7.5, 10 mM MgCl_2_, 10 mM dithiothreitol (DTT), and 10 mM ATP. For radioactive IVK assays, we added 10µCi of radiolabeled [γ-^32^P] ATP purchased from Perkin Elmer (cat# BLU002H250UC). The reactions were incubated for 30 min at 30 °C and subsequently were separated by SDS-PAGE, stained with Coomassie Brilliant Blue, and exposed to X-ray film for 72 h. For mass spectrometric (MS) analysis, YAP1 bands were excised after Coomassie staining and sent to the Kentucky MS facility.

### 2.10. Identification of YAP1 Phosphorylation by Mass Spectrometry

The band corresponding to YAP1 was excised and subjected to dithiothreitol reduction, iodoacetamide alkylation, and in-gel chymotrypsin digestion. Peptides were extracted, concentrated, and subjected to LC–MS/MS analysis at the University of Kentucky Proteomics Core Facility, as previously reported [32]. Briefly, LC–MS/MS analysis was performed using an LTQ-Orbitrap mass spectrometer (Thermo Fisher Scientific, Waltham, MA) coupled with an Eksigent Nanoflex cHiPLC system (Eksigent, Dublin, CA, USA) through a nano-electrospray ionization source. The peptide samples were separated with a reversed-phase cHiPLC column (75 μm × 15 cm) at a flow rate of 300 nL/min. Mobile phase A was water with 0.1% (*v/v*) formic acid, while B was acetonitrile with 0.1% (*v/v*) formic acid. The data-dependent acquisition method consisted of an Orbitrap MS scan (250–1800 m/z) with 60,000 resolution for parent ions, followed by MS/MS for fragmentation of the 10 most intense multiple charged ions. The LC–MS/MS data were submitted to a local Mascot server for MS/MS protein identification via Proteome Discoverer (version 1.3, Thermo Fisher Scientific, Waltham, MA, USA). Typical parameters used in the Mascot MS/MS ion search were chymotrypsin digestion with a maximum of two miscleavages; 10 ppm precursor ion and 0.8 Da fragment ion mass tolerances; and dynamic modifications, including cysteine carbamidomethylation, methionine oxidation, and serine/threonine/tyrosine phosphorylation. The identified phosphorylation sites were illustrated with relevant b and/or y ions labeled.

### 2.11. Western Blotting

Cells were collected and lysed by sonication in 1X RIPA lysis buffer. Protein concentration was determined using a Pierce BCA protein assay kit (Thermo Scientific, cat# 23225, Waltham, MA, USA). Samples from the lysate or co-IP or IVK assays were separated by SDS-PAGE gels and transferred to polyvinylidene fluoride (PVDF) membrane. The membrane was blocked in 5% non-fat dry milk for 1 h at room temperature and incubated with primary antibodies overnight at 4 °C. Afterwards, HRP-conjugated secondary antibodies were used to incubate the blots for 1 h at room temperature, and finally the specific proteins were detected by chemiluminescence using ECL substrates (Thermo Scientific, Waltham, MA, USA, cat# 32106) or by colorimetry using Opti-4CN substrate kit (Biorad, cat# 1708235, Waltham, MA, USA). The membrane was visualized by Biorad chemidoc imaging system (Biorad, Hercules, CA, USA, cat# 12003154). Densitometric quantifications of each blot in arbitrary units relative to the loading control are shown in Figure S5.

### 2.12. Real-time Quantitative PCR (RT-qPCR)

Total RNA was isolated using a RNeasy RNA isolation minikit (Qiagen, cat# 74104, Germantown, MD, USA) according to the manufacturer’s instructions. Complementary DNA (cDNA) was synthesized using 1μg of RNA/reaction using ProtoScript First Strand RNA synthesis reverse transcriptase and oligo (dT) primers (New England Biolab, cat# E6300L, Ipswich, MA, USA). qPCR was conducted using iQ SYBR green supermix (Biorad, cat# 1708880, Des Plaines, IL, USA) and Bio-Rad CFX96 Fast Real-Time PCR Systems. Gene expression changes were determined by ΔΔCt relative quantification method. GAPDH mRNA was used as an internal control. All values are presented as mean ± standard error mean (SEM).

### 2.13. Bioinformatics Analysis

mRNA expression analyses of TCGA patient datasets were conducted using the UALCAN online platform [33]. Oncoprints of the NEK1 and YAP1 protein level of at least more than 0.5-fold increase was generated using Cbioportal [34] from The Cancer Genome Atlas (TCGA-firehose legacy) datasets. Proteomic level of NEK1 and YAP1 based on the immunohistochemistry (IHC) analysis in different cancers were determined using the Human Protein Atlas [35] database. Representative IHC images of high-grade prostate adenocarcinoma (PRAD) and metastatic head and neck squamous cell (HNSC) carcinoma were also obtained from the Human Protein Atlas database. Volcano plot of gene enrichment correlated with NEK1 upregulation of TCGA (firehose legacy) head and neck cancer study was generated using the cBIOPORTAL web tool.

## 3. Results

### 3.1. NEK1 Regulated the Stability of YAP

**Figure 1:**
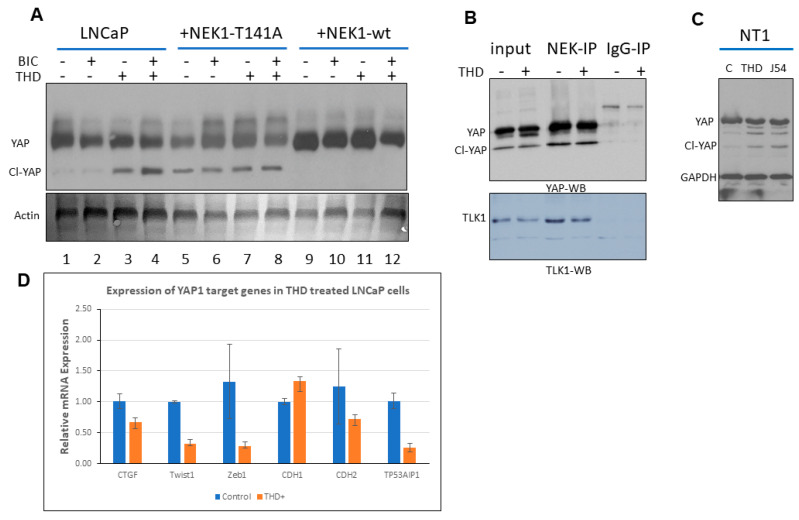
(**A**) The expression of yes-associated protein (YAP) was regulated by never in mitosis gene A (NIMA)-related kinases (NEK) activity and its upstream kinase tousled-like kinase (TLK). Overexpression of wt-NEK1 resulted in elevated YAP expression and conversely in its degradation in LNCaP cells overexpressing the dominant negative mutant NEK1-T141A. Thioridazine (THD) led to degradation of YAP in parental LNCaP cells, even after treatment with bicalutamide (BIC), which led to overexpression of TLK1B. (**B**) YAP interacted with NEK1 and was enriched upon co-immunoprecipitation. TLK1 inhibition with 10 µM THD did not affect NEK1 interaction with YAP, and thus the state of NEK1 kinase activity did not affect YAP binding. (**C**) The expression of YAP was decreased in NT1 cells treated with two different inhibitors of TLK (THD and J54), with a corresponding increase in CL-YAP products. (**D**) Expression of several typical YAP target genes in LNCaP cells treated with THD.

We have previously reported that androgen deprivation in LNCaP cells results in a strong increase in expression of TLK1B. This increase is mTOR-dependent and suppressible with rapamycin [24]. Similar results were obtained with TRAMP-C2 cells [24], and more recently in a AR+/PDX adenocarcinoma model (NSG-TM00298 [25]). This is apparently a critical survival mechanism of AS-PCa cells that implement a DDR in order to arrest in G1 upon androgen deprivation-like treatment with bicalutamide (BIC) [36]. We have recently attributed the probable mechanisms causing this DDR activation to the role played by the AR as a replication licensing factor [37] in combination with the increased expression of TLK1B, and resulting activation of the NEK1>ATR>Chk1 axis [24], which is a key target of TLK1 [23]. Additional work from our lab suggested that this may be a conserved nexus in other cellular models, in the TRAMP mice, and probably in many patients, since the specific activating phosphorylation of NEK1 by TLK1 correlates with the Gleason score [25]. While the significance of the cell cycle arrest upon unfavorable growth conditions (androgen deprivation therapy, ADT) seems clear in order to avoid mitotic catastrophe, it is still unclear how AS-PCa cells eventually adapt to ADT and reprogram to become AI (CRPC progression). Interestingly, we have previously noticed that when LNCaP cells were stably transfected with a wt-NEK1 expression vector, they rapidly (less than 1 week) became tolerant to BIC and resumed growth to form AI colonies [24]. However, this did not happen when we expressed the hypoactive T141A-NEK1 variant [23] that cannot be phosphorylated/activated by TLK1, while these cells also remained AS when injected as xenografts [24]. The rapid resumption of growth of LNCaP-NEK1 cells in the presence of anti-androgen (BIC) could not be readily explained by the implementation of the pro-survival DDR checkpoint, suggesting that NEK1 also promotes the AI conversion. On the basis of a review of the literature (see the Introduction), we suspected that NEK1 may affect the Hippo pathway, and thus we carried out a Western blotting (WB) analysis of YAP expression in LNCaP cells overexpressing wt-NEK1 or NEK1-T141A variant. The cells were also treated or not treated with BIC and thioridazine (THD), which is a rather specific inhibitor of TLKs [38]. In Figure 1A (quantitation in Figure S5), we show that overexpression of the NEK1-T141A variant results in reduced levels of YAP (lane 1 vs. 5), along with evidence of an elevated cleaved product (Cl-YAP). Decreased YAP levels and evidence of Cl-YAP were also seen in parental LNCaP cells treated with THD (+/− BIC, lane 3 and 4 vs. 1). In contrast, LNCaP cells that overexpress wt-NEK1 showed elevated expression of YAP and no evidence of Cl-YAP (lanes 9–12), where a possible mechanism is that the phosphorylation of YAP by elevated wt-NEK1 mediates a process of stabilization to counteract its degradation when TLK activity (upstream of NEK1) is suppressed with THD (lane 11 vs. 3). Furthermore, the expression of typical YAP/TEAD-dependent transcripts such as CTGF, CDH2 (N-cadherin), Twist1, and TP53AIP1 were decreased in LNCaP cells treated with THD, while in contrast, the expression of CDH1 (E-cadherin) that drives MET was slightly increased (Figure 1D).

We also show that NEK1 interacted with YAP, as it was enriched by co-IP, and their association was not altered by THD (Figure 1B, top panel; quantitation in Figure S5), indicating that the NEK1 kinase activity was independent of its ability to interact with YAP. As we previously reported [23], the same co-IP also brought down TLK1, and THD did not affect their interaction (Figure 1B, bottom panel; quantitation in Figure S5). There is a possibility that in cells, NEK1, TLK1, and YAP are in a complex, or that NEK1 interacts independently with TLK1 and YAP. In either case, TLK1 was not found to interact directly with YAP [23].

To confirm the effect of inhibition of the TLK1>NEK1 axis on the expression of YAP in a different PCa cell line, we treated Neo-TAg1 (NT1) with two different inhibitors of TLK1: THD or J54. This resulted in a reduction of YAP level and appearance of a set of cleavage products (Figure 1C; quantitation in Figure S5).

### 3.2. NEK1 KO in NeoTag1 Cells Resulted in Reduced YAP Levels and Expression of Several of Its Target Genes

**Figure 2:**
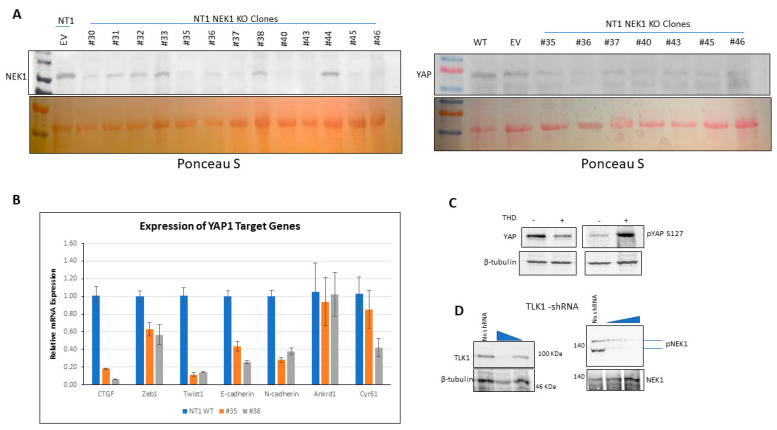
(**A**) CRISPR/Cas9-mediated loss of NEK1 resulted in reduced levels of YAP protein, possibly due to instabilization (EV = empty vector). (**B**) Expression of several typical YAP target genes is reduced in NEK1 KO clones. GAPDH mRNA was used as an internal control. (**C**) Treatment of LNCaP cells with THD, a specific inhibitor of TLKs, resulted in reduced YAP protein level and conversely in its S127 hyperphosphorylation. (**D**) Reduction of TLK1 expression via (short hairpin) shRNA transfection led to loss of pNEK1-T141.

Consistent with our initial observations that NEK1 activity is critical for YAP stabilization, we found that YAP expression was concomitantly reduced in CRISPR-mediated KO of NEK1 in the PCa line NT1 (Figure 2A; quantitation in Figure S5). Likewise, the expression of several YAP target genes (e.g., CTGF, Zeb1, Twist1) that drive Epithelial to Mesenchymal Transition (EMT) and invasiveness of these cells was suppressed in all the positive NEK1 KO clones (Figure 2B). Conversely, inhibition of TLK1 with THD, which we showed leads to reduced NEK1 activity [23], can inhibit cell migration via suppression of EMT-related genes such as Claudin1, E-cadherin, N-cadherin, Twist1, Snail3, Slug, FOXC2, MMP3, and MMP9 in Hepato Cellular Carcinoma (HCC) cells [39]. We now suggest this observation derives from reduction of YAP expression concomitant with loss of NEK1 (activity) due to inhibition of TLK. In fact, we showed in Figure 2C (quantitation in Figure S5) that YAP expression was reduced in LNCaP cells treated with THD, while conversely, pYAP(S127), which is a phospho-degron leading to its proteasomal degradation, was elevated.

In order to confirm with a genetic approach that the inhibition of TLK1 results in suppression of the pathway that leads to activation of NEK1 and subsequent stabilization of YAP, we knocked down TLK1 with shRNA in HeLa cells. Effective knockdown of TLK1 was achieved in a dose-dependent manner with the shRNA (Figure 2D; quantitation in Figure S5), and importantly, activated NEK1 levels, i.e., pNEK1(T141) were similarly suppressed. This suggests that at least in these cells, TLK1 is the principal kinase responsible for the phosphorylation and activation of NEK1—note that the T141 residue resides in the kinase domain of the protein adjacent to the activation loop [40] that we have previously shown to be important for NEK1 kinase activity [23].

### 3.3. NEK1 Phosphorylated YAP In Vitro on Several Residues

**Figure 3:**
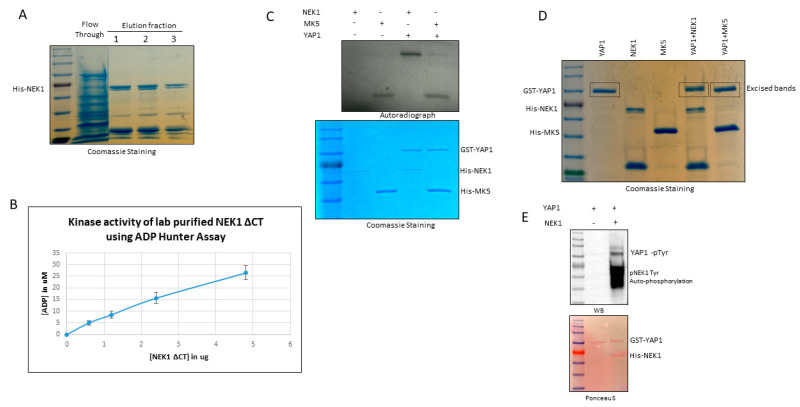
(**A**) Expression and purification of His-NEK1 kinase domain (NEK1∆CT). (**B**) NEK1∆CT was catalytically active and ATP/ADP conversion (kinase activity) was linear with the enzyme amount. (**C**) In vitro phosphorylation reactions of YAP using His-NEK1 and MK5 kinases in presence of [γ-^32^P] ATP. (**D**) In vitro phosphorylation of YAP using His-NEK1 and MK5 kinases for preparative isolation for MS determination of phosphopeptides. (**E**) His-NEK1 also phosphorylated YAP on Tyr, as demonstrated by immunoreactivity with pY antibody.

In order to determine if NEK1 could phosphorylate YAP in vitro, we first purified a recombinant His-tagged NEK1-NT fragment spanning nearly half of the entire protein (total NEK1 protein = 1258 AA, Singh et al. (2017) [ref 23]) following standard protocol and determined its catalytic activity using dephosphorylated α-casein by ADP Hunter assay (Figure 3A,B, see the Section 2). ADP hunter assay revealed that our lab-purified truncated NEK1 is catalytically active, as the incubation of increasing amounts of NEK1 resulted in corresponding ATP to ADP conversion (Figure 3B). Afterwards, we carried out a preliminary in vitro kinase (IVK) reaction by incubating purified recombinant His-tagged NEK1 with purified recombinant GST-YAP (Novus Biologicals) and [γ-^32^P] ATP. For comparison, we also carried out the IVK reaction using recombinant MK5, which was recently reported to be a novel YAP1 kinase [41]. The purity of all recombinant proteins is shown in the Coomassie Blue-stained SDS/PAGE, and the autoradiography of the gel is shown above it (Figure 3C; quantitation in Figure S5). Notably, NEK1 was capable of strongly phosphorylating YAP, even when small amounts were used (see stained gel). In contrast, MK5 (even in high amount) was a very weak kinase for YAP, if at all, although it was clearly highly active since it was capable of auto-phosphorylation (see autoradiogram) and when tested with ADP Hunter reagent.

**Figure 4:**
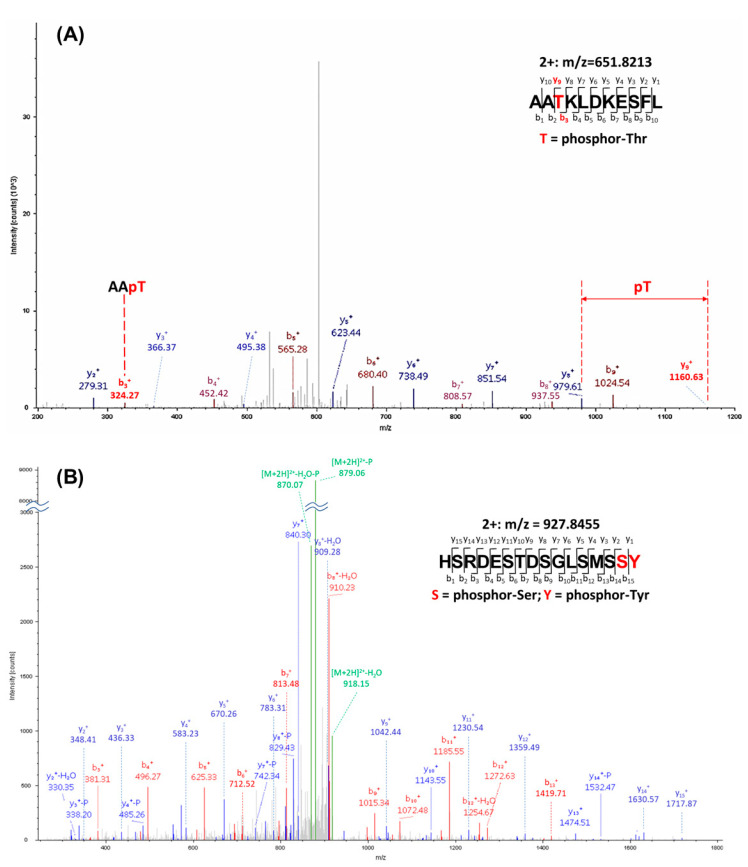
MS/MS spectra demonstrating the phosphorylation sites at T493 (**A**) and S406/Y407 (**B**) as examples of LC–MS/MS determinations.

The IVK reactions were repeated with greater amounts of proteins for preparative isolation for MS analysis for assignment of the phosphorylated residues (Figure 3D). The bands corresponding to YAP incubated with NEK1, MK5, or mock were excised. Determination of the phosphorylated peptides and assignment of the phospho-amino acids were carried out at the University of Kentucky Proteomics facility. The YAP bands were digested with chymotrypsin and analyzed with an LTQ-Orbitrap mass spectrometer. MS datasets were searched with MASCOT against a custom database containing only human YAP1 and NEK1. A synopsis of the results is that (1) when searched against YAP1 and NEK1, only YAP was detected in these samples (well separated on the gel), with 43–49% peptide coverage and protein scores of 2573-3321; (2) potential phosphorylation sites S163/S164 were detected in all three samples (including the YAP1 no kinase sample), which can be explained as a basal phosphorylated residue of recombinant YAP isolated from wheat germ; and (3) six unique phosphorylation sites were detected in the YAP_NEK1 sample: T83, T361, S366, S388, S406, Y407, or T493 (Figure 4 and Figures S1–S4; Table S1). However, no unique phosphorylation site was detected in the YAP_MK5 sample, which we now suggest is not an authentic YAP kinase. Interestingly, in the paper that purported MK5 as an important YAP kinase, the authors did not report whether they attempted to verify that MK5 can phosphorylate YAP in vitro, nor did they identify the phosphorylation target in vivo [41]. In Figure 4, we present an example of data identifying Y407 and T493, which we currently assume are the most interesting.

All of the phosphorylated residues listed in Table S1 have been reported in MS studies in cells, according to the report of Phosphosite Plus, except for S406 (putative) and T493, which, as such, are the first report of phosphorylation of these residues specifically by NEK1. The phosphorylation of Y407 (putative) should not be surprising, since NEK1 is a dual specificity kinase that was originally identified as a tyrosine kinase [42]. Note that although the MS/MS spectrum could not distinguish the exact phosphorylation site at S406 or Y407, a phospho-Tyr Western blot (Figure 3E; quantitation in Figure S5) supported the conclusion that Y407 (the only identified pTyr in the MS analysis) was phosphorylated. It is also noteworthy that the NEK1 protein was also phosphorylated on Tyr (Figure 3E), as we previously reported that it is in fact auto-phosphorylated on Y315 [23], confirming the specificity of the antiserum.

### 3.4. Bioinformatic Studies Suggest NEK1 Mediated Stabilization of YAP1 in Different Cancers

**Figure 5:**
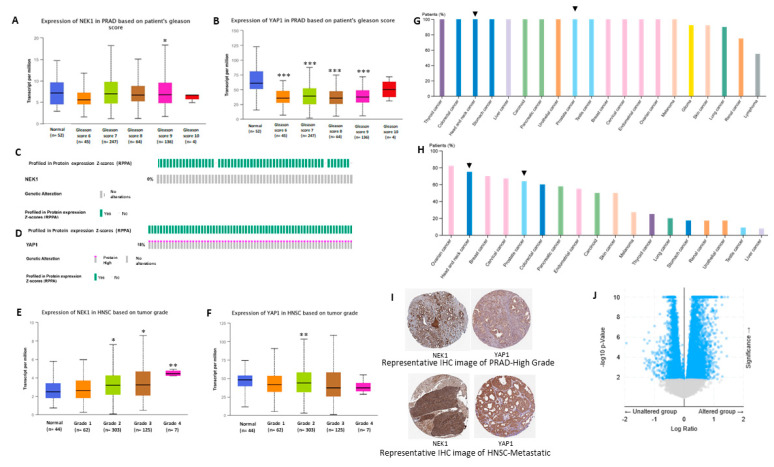
Gene expression of (**A**) NEK1 and (**B**) YAP1 of prostate adenocarcinoma (PRAD) patients on the basis of the Gleason score extracted from The Cancer Genome Atlas (TCGA) datasets using the UALCAN web tool. OncoPrint representation of the protein level alteration of (**C**) NEK1 and (**D**) YAP1 of PRAD patients by reverse phase protein array (RPPA) extracted from TCGA (firehose legacy) datasets using cBIOPORTAL online platform. Gene expression of (**E**) NEK1 and (**F**) YAP1 in head and neck squamous cell (HNSC) patients on the basis of the tumor grade extracted from TCGA datasets using the UALCAN web tool. Percentage of patients of different types of cancer with higher level of (**G**) NEK1 and (**H**) YAP1 on the basis of immunohistochemistry (IHC) staining generated using the Human Protein Atlas database. Staining intensity correlated with the color code. Deeper color represents high staining intensity. (**I**) Representative IHC images of NEK1 and YAP1 of high-grade PRAD (top panel) and metastatic HNSC samples (bottom panel). (**J**) Volcano plot of gene enrichment analysis based on NEK1 overexpression in HNSC patients extracted from TCGA (firehose legacy) datasets using cBIOPORTAL online platform. * represents *p* < 0.05, ** represents *p* < 0.005, and *** represents *p* < 0.0005. All comparisons were with the normal tissue.

We analyzed mRNA expression of both NEK1 and YAP1 in prostate adenocarcinoma (PRAD) and head and neck squamous cell (HNSC) carcinoma patients from TCGA datasets using the UALCAN online platform. In PRAD, no significant alteration in mRNA expression of NEK1 was observed (Figure 5A), while YAP1 mRNA level was consistently downregulated with respect to the tumor Gleason score (Figure 5B). However, reverse phase protein array (RPPA)-based protein profiling of NEK1 and YAP1 in PRAD patients from TCGA datasets revealed upregulation of YAP1 level (Figure 5D), but no change in NEK1 protein level (Figure 5C). In addition, proteomic analysis based on immunohistochemistry (IHC) data from the Human Protein Atlas web server revealed a higher protein level of NEK1 and YAP1 in high-grade PRAD patients (Figure 5G,H). Representative IHC analysis revealed intense staining of both NEK1 and YAP1 in high-grade PRAD compared to normal prostate tissue (Figure 5I). This supports our hypothesis of NEK1 implication in YAP1 protein stabilization/accumulation in advanced PCa, despite YAP1 transcript downregulation.

Similarly, in head and neck squamous cell carcinoma (Figure 5F), glioblastoma, and other cancers (data not shown), there was no significant upregulation of YAP1 mRNA expression; nonetheless, YAP1 protein level was elevated in high-grade metastatic tumors (Figure 5). Moreover, gene set enrichment analysis significantly correlated NEK1 expression with several YAP1 target genes such as Zeb1, BirC2, BirC6, Ankrd11, and ARID1B (Figure 5J; Table 1). Overall, these data suggest NEK1 increases YAP1 level by reducing YAP1 protein turnover rate in different cancers.

## 4. Discussion

During studies aimed at elucidating the process of ADT adaptation of AS PCa cell (initially in LNCaP), which proceeds through a process of activating the DDR and increased activity of the kinases TLK1B and NEK1 [11,24,25], we made the observation that overexpression of wt-NEK1, but not the hypoactive NEK1-T141A variant that cannot be activated by TLK, resulted in a rapid adaptation to bicalutamide and formation of AI colonies. From a review of the literature on the process of AI conversion of LNCaP and other studies of CRPC progression, we suspected the involvement of Hippo pathway deregulation and, in particular, YAP-driven gene expression (for a recent review, see [43]). Moreover, Yim et al. reported that NEK1 can phosphorylate TAZ and regulates its turnover rate [18]. Since YAP1 and TAZ are two highly homologous proteins that possess several conserved phospho-residues, we set out to investigate the protein level of YAP in LNCaP overexpressing wt-NEK1 and the T141A mutant in conjunction with a TLK inhibitor (THD) to suppress the activating phosphorylation of NEK1. Interestingly, we observed an increased degradation of YAP in cells overexpressing NEK1-T141A mutant or parental LNCaP treated with THD, in contrast to elevated level of YAP (and no degradation) in cells that overexpress wt-NEK1 (Figure 1). Furthermore, treatment of LNCaP cells with THD resulted in downregulated expression of several YAP-dependent transcripts (Figure 1D). As an indication that this is in fact a general phenomenon in PCa, increased degradation of YAP1 after inhibition of the TLK1>NEK1 axis with THD or J54 was independently verified in mouse NT1 cells (Figure 1C). In addition, genetic depletion of NEK1 resulted in YAP1 loss and YAP1 target gene downregulation in NT1 cells (Figure 2). It should be noted that YAP is a generally unstable protein whose turnover rate is strongly regulated by multiple stabilizing [44] or de-stabilizing phosphorylation events controlled by multiple kinases (see [19,20,26] for some reviews). Large tumor suppressor 1 and 2 (LATS1/2), the core kinases of the Hippo signaling pathway, can phosphorylate YAP1 on Ser127 residue, which creates a binding site for 14-3-3 proteins. The 14-3-3 binding of YAP leads to the cytoplasmic sequestration of YAP [45,46]. Sequential phosphorylation by LATS1/2 on YAP Ser397 primes it for further phosphorylation by Casein Kinase CK1δ/ε on Ser400 and Ser403, which creates a phosphodegron motif for (Skp Cullin F box) β-TrCP/SCF E3 ubiquitin ligase-mediated proteasomal degradation [47]. Recent findings also identify factors such as NR4A1 (nuclear receptor superfamily) that regulate the 14-3-3 interaction with YAP1 and promote its ubiquitination and degradation [48]. Several other kinases independent of the Hippo pathway can regulate the stability of YAP1 protein. For instance, nuclear Dbf2-related kinase (NDR1/2) can also phosphorylate YAP on Ser127 residue and can promote its cytoplasmic retention, thereby negatively regulating YAP stability [49]. Evidence suggests that the protein kinase B/AKT can also phosphorylate YAP on Ser127 residue, leading to binding of 14-3-3 and cytoplasmic retention [45]. In contrast, several members of the Src family of kinases such as Src, Yes, and c-Abl can positively regulate YAP stability. c-Abl/Src/Yes are known to phosphorylate YAP on Tyr357 residue, which results in the nuclear translocation and, hence, stabilization of YAP [44,50,51]. Moreover, Ras-associated factor isoform 1C (RASSF1C) is known to promote tyrosine phosphorylation of YAP1 (Tyr357) through activated Src (pTyr416) and cause nuclear localization of YAP1 [52]. Similarly, mitogen-activated protein kinases such as c-Jun-N-terminal kinases (JNK1/2) are also reported to be YAP kinases that phosphorylate YAP on Ser317 and Thr362, promoting YAP nuclear translocation and stabilization [53]. Thus, post-translational modifications such as phosphorylation determine YAP turnover rate and activity.

Therefore, we propose the phosphorylation of Y407 as one potential mechanism of YAP stabilization and increased transcriptional output, although the other 5-phosphorylation sites could be equally important (Figure 4 and Figures S1–S4; Table S1). There are examples in YAP and TAZ where phosphorylation of some residues impairs ubiquitination and subsequent proteasomal degradation, as in one example, phosphorylation of S128 by NLK competed for the destabilizing LATS1-dependent S127 phosphorylation [54]. However, we currently favor a pY407-related mechanism based on the equivalent pY316 of TAZ, where it was shown that the phosphorylation of that residue, reportedly by c-Abl, was necessary to mediate its interaction with the transcription factor NFAT5 [55]. This was implicated in an inhibitory pathway of NFAT5—a major osmoregulatory transcription factor—during hyperosmotic stress. Similarly, JNK1/2-mediated phosphorylation of YAP1 on Ser317 and Thr362 promotes YAP’s ability to bind and stabilize both pro-apoptotic p73 and pro-proliferative ΔNp63α in different cell types [53,56]. We think that, likewise, pY407 promotes the interaction of YAP with some of its transcriptional partners, and hence promotes its nuclear translocation, function, and stabilization, away from cytoplasmic degradation. Importantly, while the phosphorylation of Y407 was identified in proteomic studies [57], to our knowledge, the kinase responsible for it has not been reported.

Resistance to androgen deprivation therapy (ADT) promotes androgen-independent growth and proliferation of PCa cells, which requires efficient DNA damage response (DDR) and repair mechanisms, activation of compensatory signaling pathways, transcription factors, and co-factors to drive castration resistance. Findings from our lab and others suggest that ADT activates the TLK1-NEK1 signaling pathway that promotes PCa progression by activating the DDR [11,24]. Hyper-activation of NEK1 may also lengthen G2/M checkpoints, which provides the cells sufficient time to repair their damaged DNA after ADT or radiation therapy [7,58]. However, DDR alone may not be able to induce androgen-insensitive growth of PCa cells. Thus, we hypothesize that TLK1-NEK1 may be implicated in some other signaling pathway, leading to AI growth. YAP1 is a major oncoprotein that drives many different types of malignancies, including PCa [59], head and neck cancer [59], gastric cancer [60], colon cancer [60], thyroid cancer [61], lung cancer [62], ovarian cancer [63], and liver cancer [64]. NEK1-mediated phosphorylation of YAP1 (most probably on Tyr407 and/or Thr493) may induce a conformational change that counteracts the sequential phosphorylation by LATS1/2 and CK1δ/ε and subsequently protects YAP from proteasomal degradation. Moreover, Tyr407 lies on the transcriptional activation domain of YAP1, which may increase its interaction affinity to its assigned transcriptional factors [65]. Ectopic YAP expression was reported to drive LNCaP cells from androgen-sensitive to androgen-insensitive states [19]. Reducing the turnover rate will increase cellular accumulation of YAP, which can enable its oncogenic properties to drive castration resistance by several mechanisms. Previous studies reported that YAP can mediate PI (3)K-mTOR signaling and activate AKT [66,67,68]. Activation of mTOR will lead to enhanced translation of TLK1B that can, in turn, increase YAP1 phosphorylation through TLK1-NEK1 nexus. This suggests a positive feed-forward mechanism for YAP accumulation. Elevated YAP can also activate ERK that will promote cell proliferation in absence of AR signaling. Kuser-Abali et al. reported that AR and YAP can interact, and this interaction contributes to the switch from androgen-dependent to castration-resistant phenotype [27]. Overexpression of YAP can also regulate the expression of AR target genes, including PSA, NKX3.1, PGC-1, and KLK2, which suggests that YAP may control AR activity. YAP Tyr407 phosphorylation could increase the binding affinity of AR and AR ligand-insensitive variant AR-V7, thus contributing to androgen refractory growth of PCa cells. Therapy-induced YAP overexpression may also induce EMT activation by upregulating EMT-specific genes. Increasing the stemness of PCa cells can be another mechanism by which stabilized YAP can promote castration-resistant growth of PCa cells, which will further contribute to chemo-resistance of cancer cells [69]. Our bioinformatics analyses also suggested a link between NEK1 and YAP1 in different cancers (Figure 5). YAP1 protein level is abundant in high-grade PCa tumors, despite the progressive downregulation of YAP1 mRNA expression. Other groups also reported that YAP protein is positively correlated with the Gleason score, consistent with the findings of our bioinformatics analysis [70]. We propose that the signaling of TLK1>NEK1-mediated YAP phosphorylation and stabilization contributes not only to PCa progression, but also many other cancers. Importantly, we found a correlation between increased phosphorylated NEK1(T141) in relation to the Gleason score [25] and YAP1 protein expression, whereas the mRNA for YAP1 actually decreased (Figure 5), consistent with our model of post-transcriptional protein stabilization.

## 5. Conclusions

YAP’s transcriptional activity and degradation is mainly regulated by phosphorylation through several kinases dependent and independent of the Hippo pathway. Using small molecule inhibitors against YAP cannot completely abolish YAP transcriptional activity and is not very effective in treating YAP-driven cancers. Inhibitors such as verteporfin that can disrupt the YAP–TEAD interaction, but still cannot result in complete inhibition, as YAP can bind with other transcription factors such as TEF, SMADs, or TBX5. The majority of YAP kinases negatively regulate YAP by promoting its nuclear egress or degradation; however, NEK1 is found to stabilize YAP protein by phosphorylating it on several residues. Thus, targeting NEK1 or the TLK1–NEK1 axis can bring about therapeutic benefits in the clinical management of YAP-driven malignancies.

## Figures and Tables

**Table 1:** Some of the YAP target genes significantly upregulated with NEK1 upregulation in head and neck squamous cell (HNSC) carcinoma (TCGA, firehose legacy) analyzed using cBIOPORTAL.

Gene Name	Mean Log2 mRNA Expression ± SD in NEK1-Overexpressed Group	*p*-Value	*q*-Value
*Zeb1*	8.57 ± 1.00	3.68 × 10^−7^	5.153 × 10^−6^
*Zeb2*	8.80 ± 1.05	7.237 × 10^−6^	6.617 × 10^−5^
*Ankrd36B*	4.33 ± 0.93	1.410 × 10^−4^	8.367 × 10^−4^
*Ankrd11*	11.55 ± 0.49	1.159 × 10^−3^	4.984 × 10^−3^
*BirC2*	10.46 ± 0.96	0.0187	0.0498
*BirC6*	11.13 ± 0.49	3.91 × 10^−12^	2.80 × 10^−10^
*HoxB3*	6.44 ± 2.11	0.0169	0.0458
*ARID1B*	10.84 ± 0.46	4.14 × 10^−10^	1.57 × 10^−8^
*WSB2*	11.05 ± 0.45	2.016 × 10^−4^	1.136 × 10^−3^
*CAT*	10.01 ± 0.68	3.621 × 10^−4^	1.872 × 10^−3^
*ABCB1*	5.11 ± 1.45	0.0111	0.0327
*PTX3*	5.33 ± 2.12	1.994 × 10^−4^	1.126 × 10^−3^

## Supplementary Materials

The following are available online at https://www.mdpi.com/2072-6694/12/12/3666/s1: Figure S1: MS/MS spectrum of the phosphor-peptide N_70_AVMNPKTANVPQTVPMRL_88_ to determine pT83, Figure S2. MS/MS spectrum of the phosphor-peptide R_161_QSSFEIPDDVPLPAGW_177_ to determine pS163/pS164, Figure S3. MS/MS spectrum of the phosphor-peptide A_347_LRSQLPTLEQDGGTQNPVSSPGmSQEL_374_ to determine pT361/pS366, Figure S4. MS/MS spectrum of the phosphor-peptide R_375_TMTTNSSDPFLNSGTY_391_ to determine pS388, Figure S5. Densitometry of the original uncropped blots and gel images with their respective numbers from the main figures, Table S1. Assigned phosphorylated sites.
