# Supplementary material for: NEK1 Phosphorylation of YAP Promotes Its Stabilization and Transcriptional Output
Source: Cancers (Basel). 2020 Dec 7;12(12):3666. doi: 10.3390/cancers12123666 (PMC7762262; doi:10.3390/cancers12123666)
Supplement: Supplementary file 1 [file cancers-12-03666-s001.pdf]

Supplementary Materials: NEK1 Phosphorylation of YAP Promotes its Stabilization and Transcriptional Output

Md Imtiaz Khalil, Ishita Ghosh, Vibha Singh, Jing Chen, Haining Zhu and Arrigo De Benedetti

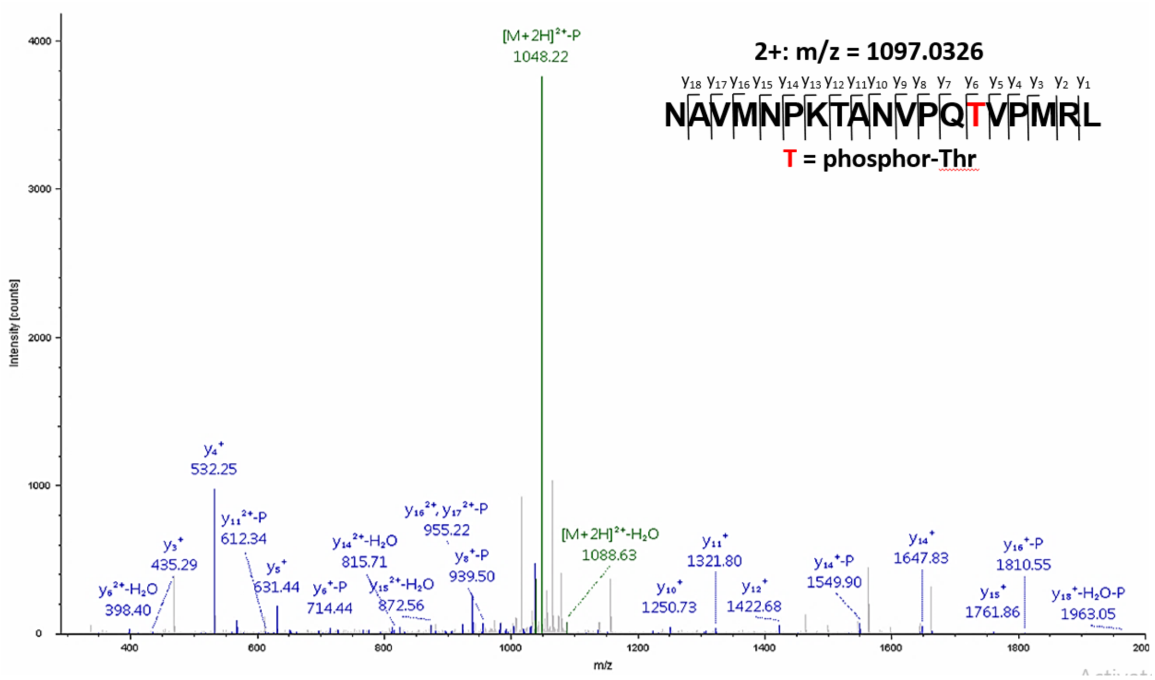

Figure S1. MS/MS spectrum of the phosphor-peptide N70AVMNPKTANVPQTVPMRL88 to determine pT83.

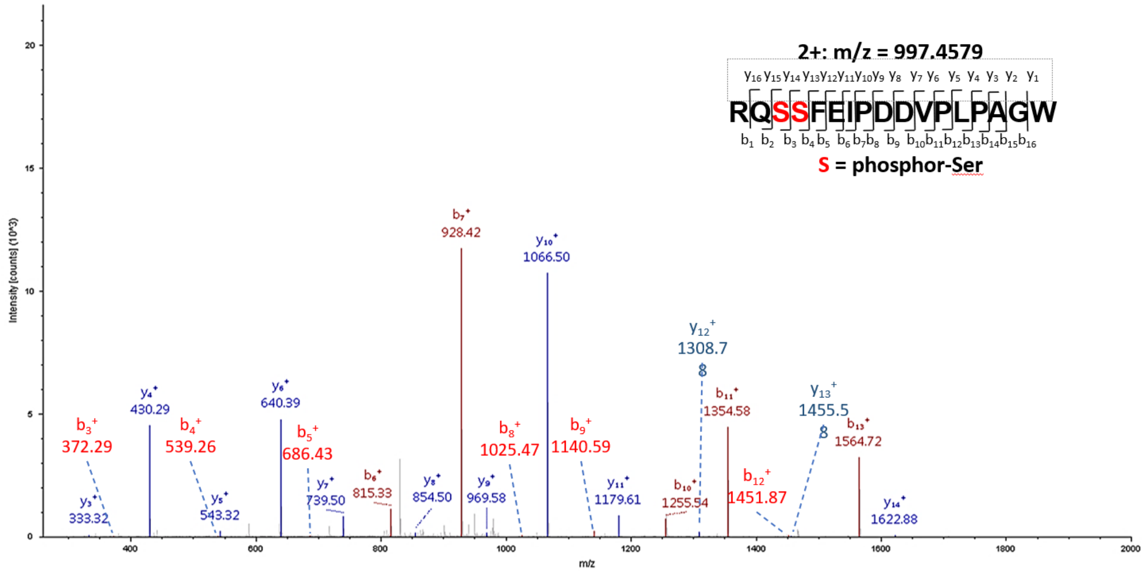

Figure S2. MS/MS spectrum of the phosphor-peptide R161QSSFEIPDDVPLPAGW177 to determine pS163/pS164. It is noted that this phosphor-peptide was detected in all 3 sample. It is noted that the MS/MS spectrum cannot distinguish pS163 or pS164.

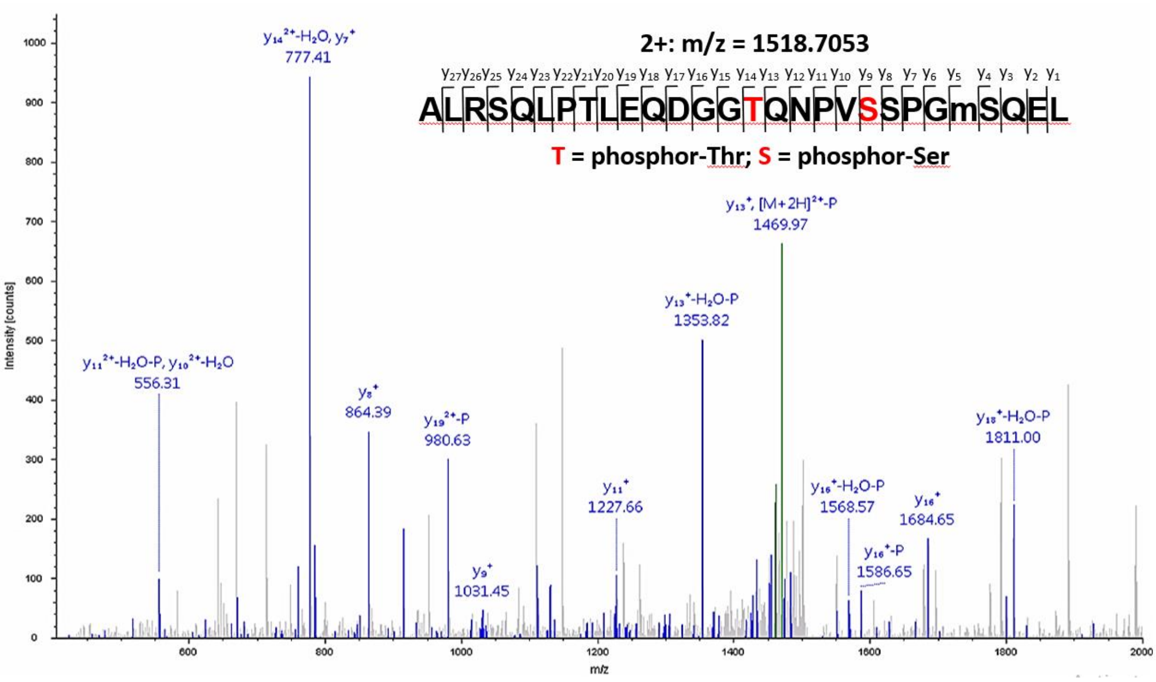

Figure S3. MS/MS spectrum of the phosphor-peptide A_{347}LRSQLPTLEQDGGTQNPVSSPGmSQEL$_{374}$ to determine pT361/pS366. It is noted that the MS/MS spectrum cannot distinguish pT361 or pS366.

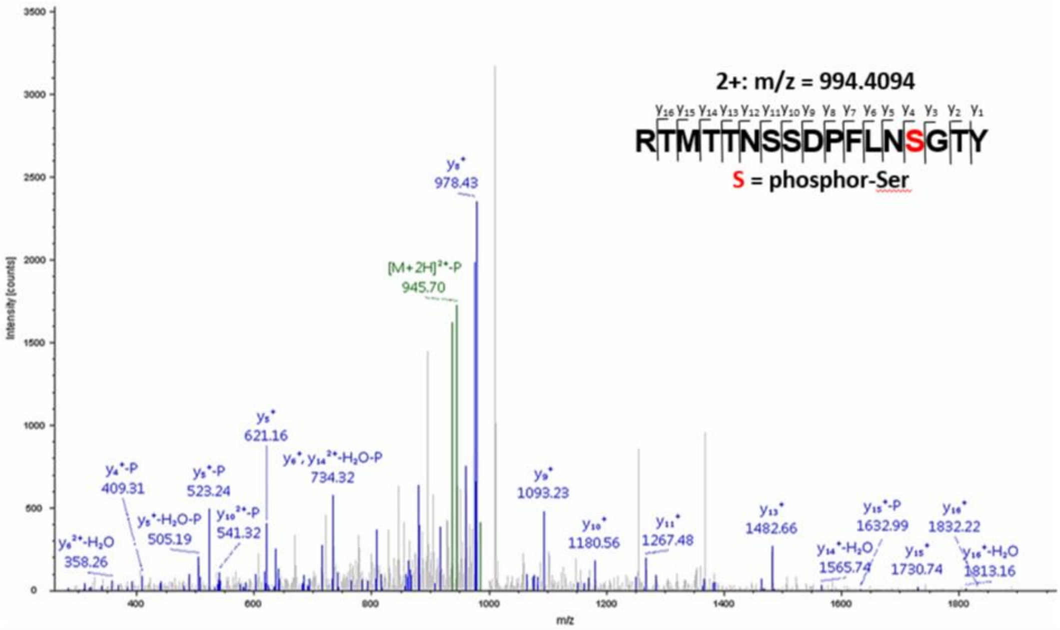

Figure S4. MS/MS spectrum of the phosphor-peptide R_{375}TMTTNSSDPFLN**S**GTY$_{391}$ to determine pS388.

Fig1A_LNCaP_YAP-orig-quantitated

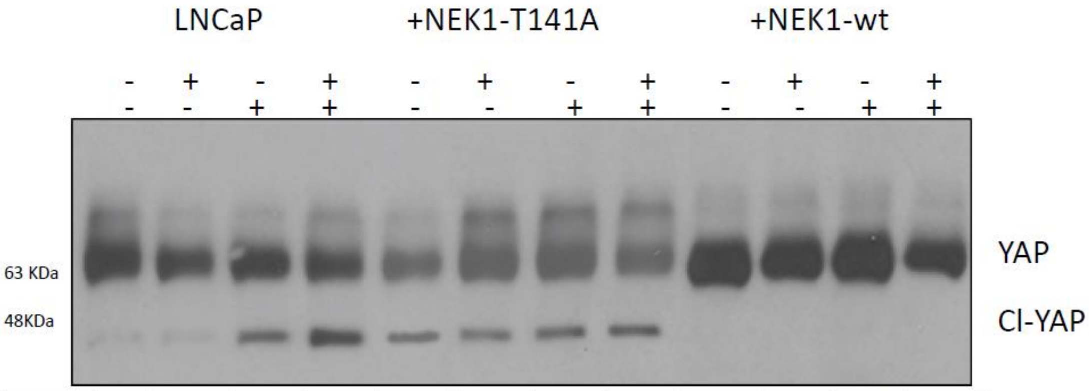

AU-YAP	1	0.63	0.58	0.53	0.43	0.61	0.53	0.35	1.33	0.79	1.27	0.74
AU-Cl-YAP	1	0.49	3.73	7.39	3.31	5.64	5.90	4.47	0	0	0	0

Fig1B_ NEK1/YAP co-IP

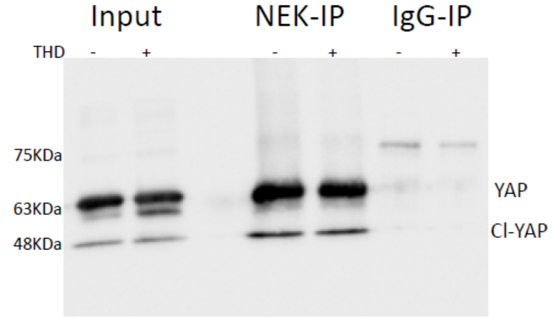

YAP	1	1.06	-	1.67	1.47	0.04	0.01
Cl-YAP	1	0.98	-	3.14	3.95	0.06	0.03

Fig1B_ NEK1/TLK1 co-IP

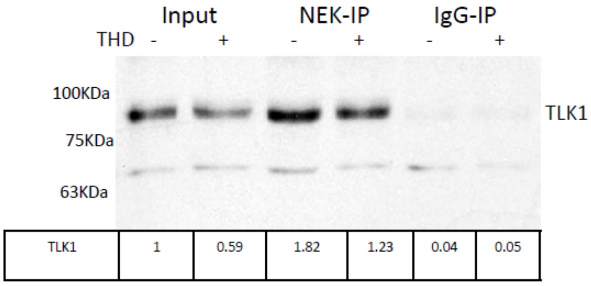

TLK1	1	0.59	1.82	1.23	0.04	0.05

Fig1C_NT1_YAP

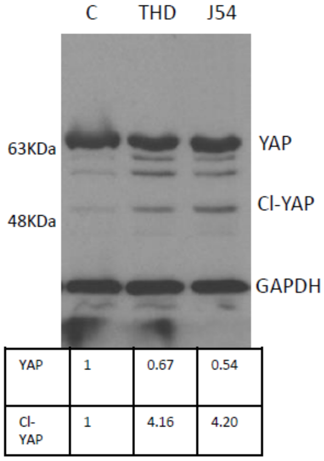

YAP	1	0.67	0.54
Cl-YAP	1	4.16	4.20

Figure 2A (Left Panel): NT1 NEK1 KO

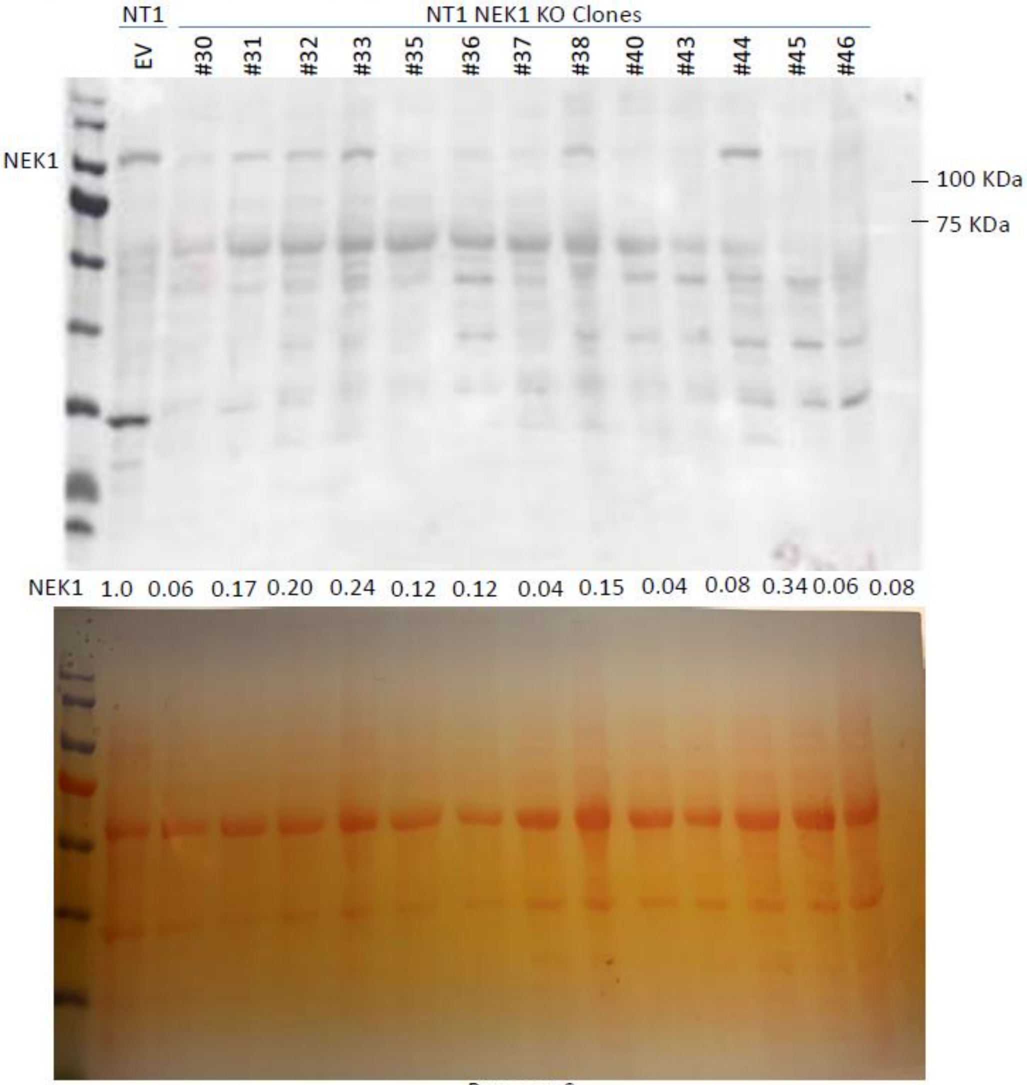

Ponceau S.

Figure 2A (Right Panel): YAP1 level in NT1 NEK1 KO cells

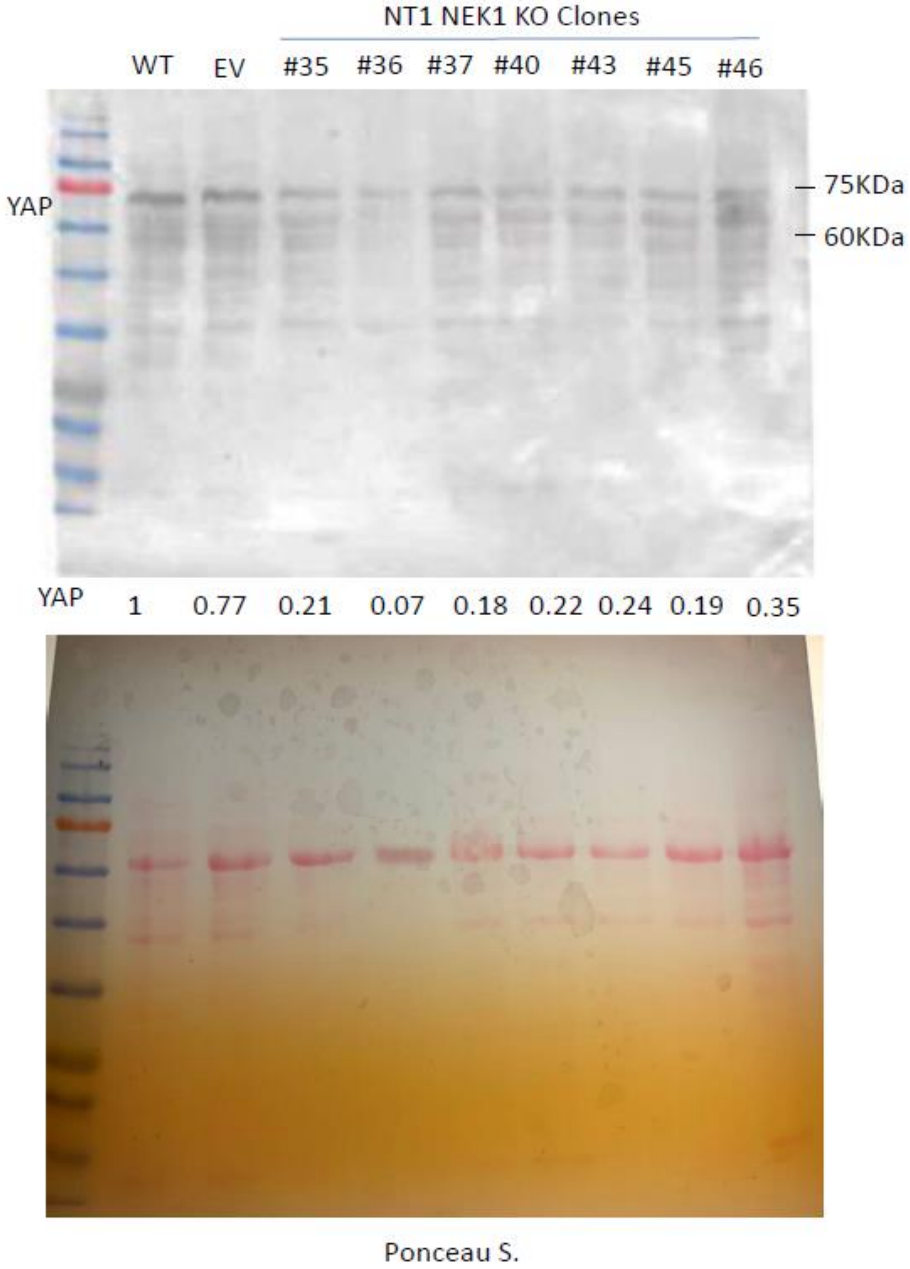

Figure 2C left panel:
YAP level upon THD treatment

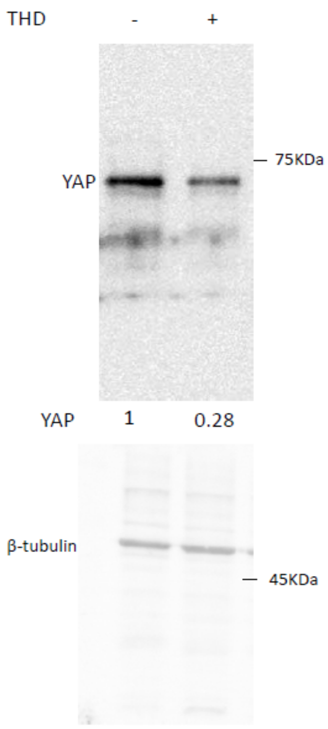

Figure 2C Right panel:
pYAP level upon THD treatment

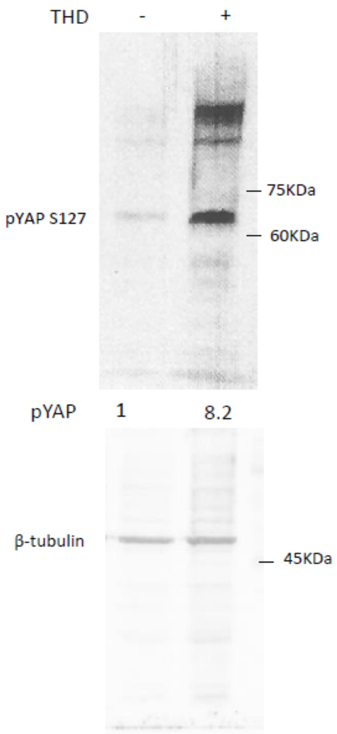

Figure 2D left panel:
TLK1 knockdown with shRNA

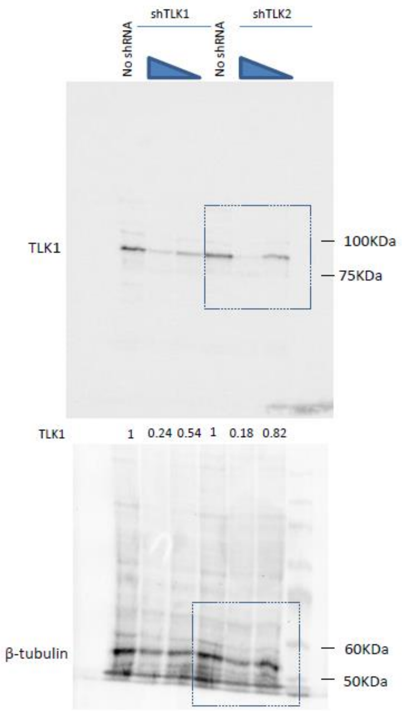

Figure 2D right panel: pNEK1 level in TLK1 KD cells

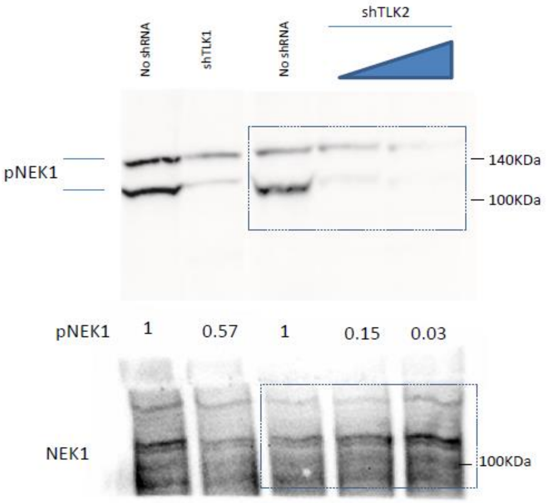

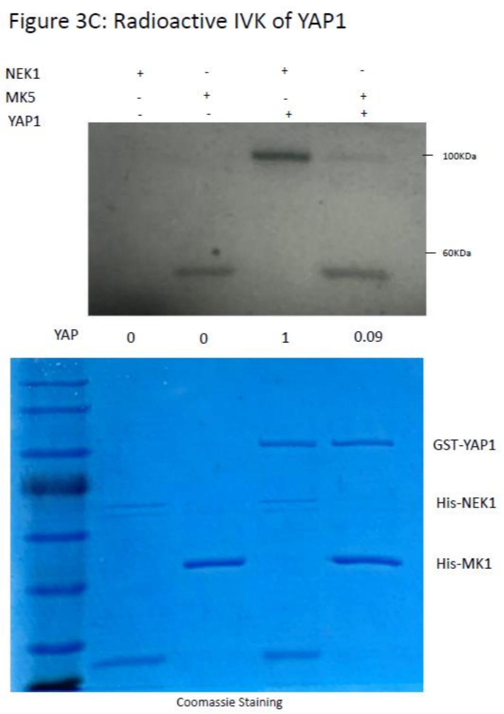

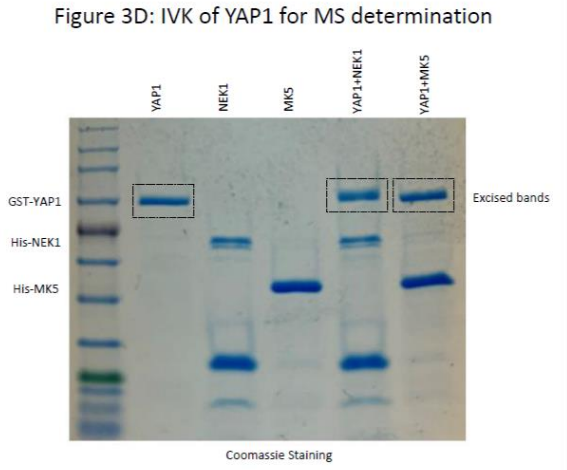

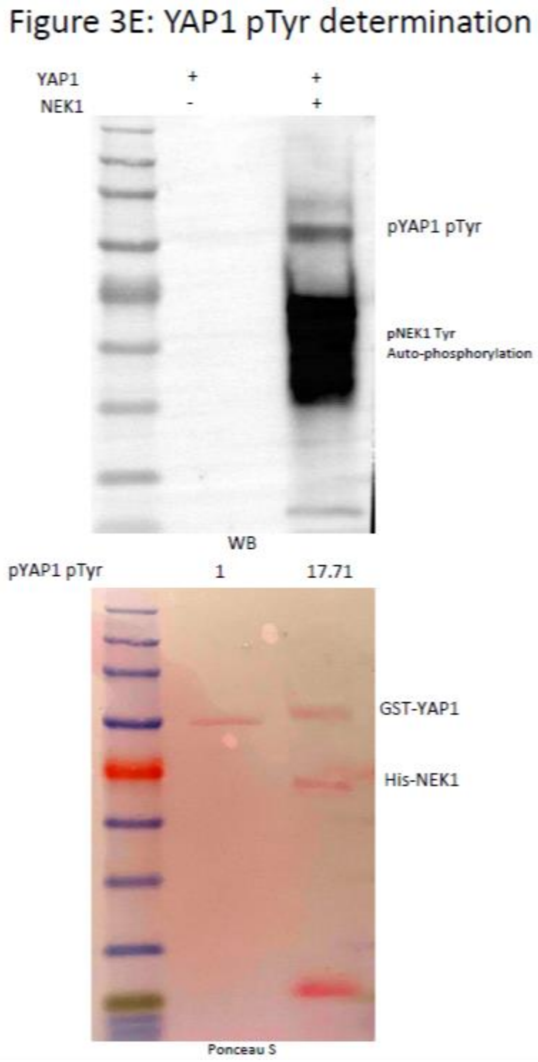

Figure S5. Densitometry of the original uncropped blots and gel images with their respective numbers from the main figures.

Table S1. Assigned phosphorylated sites.

Phosphorylated Sites	Peptides	YAP1	YAP1 + NEK1	YAP1 + MK5
T83	N_{70}AVMNPKTANVPQTVPMRL_{88}	N	Y	N
* S163/S164	R_{161}QSSFEIPDDVPLPAGW_{177}	Y	Y	Y
* T361/S366	A_{347}LRSQLPTLEQDGGTQNPVSSPGmSQEL_{374}	N	Y	N
S388	R_{375}TMTTNSSDPFLNSGTY_{391}	N	Y	N
* S406/Y407	H_{392}SRDESTDSGLSMSSY_{407}	N	Y	N
T493	A_{491}ATKLDKESFL_{501}	N	Y	N

* Cannot tell which site is preferred based on the MS/MS spectrum.
